# Intelligibility in Context Scale: Growth Curves for Typically Developing English-Speaking Children Between Ages 2;6 and 9;11

**DOI:** 10.1044/2023_AJSLP-22-00392

**Published:** 2023-06-30

**Authors:** Jennifer U. Soriano, Tristan J. Mahr, Paul J. Rathouz, Katherine C. Hustad

**Affiliations:** a Department of Communication Sciences and Disorders, University of Wisconsin–Madison; b Waisman Center, University of Wisconsin–Madison; c Department of Population Health, Dell Medical School, The University of Texas at Austin

## Abstract

**Purpose:**

The aim of this study was to quantify the clinical utility of the Intelligibility in Context Scale (ICS) English version by characterizing the growth patterns of the ICS composite scores and seven ICS individual item scores of typically developing American English–speaking children.

**Method:**

Parents of 545 typically developing children aged 2;6–9;11 (years;months) completed the ICS. Using a proportional odds model, we regressed ICS composite scores on age and computed for model-estimated mean and lower quantile ICS composite scores. Logistic regression and proportional odds modeling were utilized to quantify the relationship of individual ICS items and age.

**Results:**

ICS composite scores of typically developing children changed with age, but change was small and incremental, with scores compressed between 3 and 5 across the range of ages. An average child (i.e., on the 50th percentile) is expected to have an ICS composite score of 4 beginning at 3;0 and an ICS composite score of 5 by 6;6. On average, parents gave different intelligibility ratings based on communicative partners, and the rating differences between communicative partners decreased with age.

**Conclusions:**

Given that ICS scores increase with age, the expected score for average children also increases. A child's age is a main factor for interpreting ICS scores.

Speech intelligibility is integral to spoken communication and has been described as an index of functional oral communication (Kent, 1992; Kent et al., 1994; Yorkston & Beukelman, 1978). It is an important clinical metric for children who are at risk for or who have speech disorders (Gordon-Brannan & Hodson, 2000; Hustad & Borrie, 2021; Kent et al., 1994; Miller, 2013; Schiavetti, 1992). Intelligibility measures provide an index of functional speaking ability and may point to a need for intervention to improve functional speech. In addition, intelligibility-based metrics can be used to set a functional speech improvement target (e.g., achieving a specific level of intelligibility as an intervention goal) and for monitoring intervention progress. Marked reduction in speech intelligibility can lead to pervasive limitations in social and academic participation (McCormack et al., 2009). Thus, improving speech intelligibility that is below age expectations is an important objective for speech intervention (Hodge & Whitehill, 2010; Hustad & Borrie, 2021; Hustad et al., 2021; Lee, 2019). Having a good understanding of speech intelligibility, especially in a developmental context, is indispensable in speech-language pathology.

Various methods have been developed to measure speech intelligibility, such as orthographic transcription, closed-set word identification, phonetic indices, and scaling methods. Previous literature has described the benefits and caveats of each method (Kent et al., 1994; Lagerberg et al., 2021; Miller, 2013). Regardless of the method of measurement, age-based normative standards for the acquisition of intelligible speech in children are critically important to the identification of atypical performance.

## Transcription Intelligibility

Recent research has provided foundational knowledge on speech intelligibility growth trajectories based on transcription intelligibility methods for children between the ages of 2 and 10 years (Hustad et al., 2020, 2021; Schölderle et al., 2021). Transcription intelligibility measures involve recording speech samples from a child, preparing a master transcript of the target words or utterances produced by the child, and having naïve listeners orthographically transcribe the speech recording. Speech intelligibility is measured as the proportion of words correctly transcribed by listeners (i.e., correct words divided by the total words in the master transcript). Hustad et al. (2021) studied single-word and multiword intelligibility of English-speaking children aged 2;6–9;11 (years;months). Their results showed that intelligibility grows rapidly in the preschool years and continues to improve throughout middle childhood. For average children (50th percentile), intelligibility growth was steepest around the age of 2;9. Between-child variability was very large, especially during the preschool years, but decreased with age. Intelligibility continued to improve with age through 10 years. Similarly, Schölderle et al. (2021) examined multiword intelligibility growth of German children aged 3–9 years and had comparable findings. Note that, for both of these studies, the authors used a structured and controlled experimental measurement paradigm for both children and listeners. Therefore, generalization to real-world communication situations involving spontaneous speech and interaction between partners must be made with caution.

## Intelligibility in Context Scale

Rating scales have also been widely used for measuring intelligibility, both clinically and in research studies (Schiavetti, 1992). Rating scales involve the use of a Likert scale or a visual analog scale marked with interval ratings (Miller, 2013). Listeners are asked to estimate a child's intelligibility using the scale of interest. For instance, the Intelligibility in Context Scale (ICS; McLeod et al., 2012) asks parents to rate (on a 5-point scale) how well different partners are able to understand their child's speech during daily communication. As a rating scale, short completion time and ease of administration are clinical advantages of the ICS (Miller, 2013; Pascoe & McLeod, 2016). The ICS includes seven questions specifying different communication partners including parents themselves, immediate family, extended family, friends, acquaintances, teachers, and strangers. The ratings of the seven individual items are averaged to obtain an overall score (i.e., composite score). Research has shown that the extent to which listeners are familiar with a child's speech can influence their intelligibility ratings, with more familiar listeners giving higher ratings (Flipsen, 1995; Van Doornik et al., 2018). Thus, another advantage of using the ICS is an estimation of a child's speech intelligibility with listeners who vary in familiarity (McLeod et al., 2012). The ICS has been recommended as a screening tool for children who may have speech disorders (McLeod et al., 2015), as one of the speech outcome measures of a standard set of cleft palate–specific outcome measures (Apon et al., 2021), and as one of the procedures for a holistic assessment of speech abilities (Cronin et al., 2020; Pascoe & McLeod, 2016; Sakash et al., 2021).

The English version of the ICS was validated on 120 Australian English–speaking children aged 3;11–5;8 (McLeod et al., 2012) and on 803 Australian English–speaking children aged 4;0–5;5 (McLeod et al., 2015). The second validation study was conducted on 180 children who were typically developing, 576 children whose parents or teachers were concerned about their speech production, and 47 children with speech-language disorders (McLeod et al., 2015). Results showed high internal reliability. The average ICS composite score was 4.4 of 5.0 (*SD* = 0.7) across the whole sample (i.e., including typically developing children, children with parent/teacher concerns regarding their speech, and children with speech–language disorders). In addition, parent ratings for different communication partners were statistically different, with the ratings for themselves being the highest and the ratings for strangers being the lowest across the whole sample. The mean ICS composite scores of children whose parents did not express concerns (*M* = 4.6) and children whose parents did express concerns (*M* = 3.9) were used to determine a cutoff score to identify children who may have a speech disorder. McLeod et al. (2015) recommended a cutoff score of 4.6 (i.e., children with ICS scores below 4.6 should be referred for further evaluation), which had a sensitivity of 0.82 and a specificity of 0.58. This cutoff score is high relative to other recommended cutoff scores reported by other studies on other languages (Kok & To, 2019; Leon et al., 2021; Lousada et al., 2019; Ng et al., 2014).

A considerable amount of research has focused on evaluating different psychometric properties of translated versions of the ICS and describing the ICS composite scores of typically developing children, children whose parents had concerns about speech, and/or children with speech-language disorders (Hopf et al., 2017; Kim et al., 2016; Kok & To, 2019; Lagerberg et al., 2021; Le et al., 2021; Lee, 2019; Leon et al., 2021; Lousada et al., 2019; Neumann et al., 2017; Ng et al., 2014; Phạm et al., 2017; Piazzalunga, Salerni, Ambrogi, et al., 2020; Piazzalunga, Salerni, Limarzi, et al., 2020; Washington et al., 2017). Mean ICS composite scores reported from studies that included children who speak English and other languages are summarized in Appendix Table A1. Across studies, mean ICS composite scores for typically developing children aged 2;0–10;5 varied from 4.03 to 4.81 (of a possible 5.0), indicating that children's perceived intelligibility was very high, even for young children.

Only a few studies have reported the range of ICS composite scores of typically developing children; however, none of the existing studies included children who spoke English (Hopf et al., 2017; Piazzalunga, Salerni, Ambrogi, et al., 2020; Piazzalunga, Salerni, Limarzi, et al., 2020; Van Doornik et al., 2018). Across these studies, the range of ICS composite scores was between 2.86 and 5 for children aged 3;0–10;5. On the other hand, mean ICS composite scores for children with speech-language disorders or whose parents were concerned about their speech production varied from 3.62 to 4.39, indicating an array of scores that overlap to some extent with those from typically developing children. The range of ICS composite scores for children with speech-language disorders was wider compared with typically developing children and was between the full range of possible ICS composite scores (i.e., 1–5) for children aged 3;0–10;1. (Burton et al., 2019; Lagerberg et al., 2019; Seifert et al., 2021; Van Doornik et al., 2018).

Although the ICS is a widely used clinical tool, available normative data for English-speaking children are limited to Australian English–speaking children aged 4;0–5;5 (McLeod et al., 2015). Other available data sets that describe ICS composite scores for specific age groups are on bilingual children speaking English and Jamaican Creole aged 3;3–6;3 (Washington et al., 2017), Cantonese-speaking children aged 2;4–6;9 years (Kok & To, 2019), European Portuguese–speaking children aged 3;10–6;2 (Lousada et al., 2019), Italian-speaking children aged 3;0–5;11 (Piazzalunga, Salerni, Ambrogi, et al., 2020), Swedish-speaking children aged 3;2–9;2, (Lagerberg et al., 2021), and Vietnamese-speaking children aged 2;0–5;11 (Phạm et al., 2017). Reported mean ICS composite scores for specific age bands are summarized in Appendix Table A2. Across studies that included children who speak English and other languages, ICS composite scores increased with age, with few exceptions. For example, Lagerberg et al. (2021) reported slightly lower ICS composite scores for older children. Another common theme across studies that reported ICS composite scores for specific age bands (see Appendix Table A2) is that mean ICS composite scores of children 3 years old and above were 4 or higher for the majority of these studies.

Only three studies reported ICS composite scores at specific percentiles (see Appendix Table A3; Lagerberg et al., 2021; Lousada et al., 2019; Piazzalunga, Salerni, Ambrogi, et al., 2020). All three studies included children who speak languages other than English. ICS composite scores of children in the upper percentiles (i.e., 75th, 90th, and 95th) achieved a ceiling score of 5 at earlier ages. Children in the 50th percentile received ICS composite scores between 4 and 5. Meanwhile, ICS composite scores of children in the lower percentiles (i.e., 5th, 10th, 15th, and 25th) ranged from 3 to 4. Although these studies extend our knowledge of normative expectations for intelligibility change as measured by the ICS, we do not know the extent to which these findings are comparable in English-speaking children. Thus, additional studies on English-speaking children are necessary to establish normative expectations across a wider range of ages.

In this study, we sought to establish normative standards for ICS composite scores by developing age-based percentile growth curves for American English–speaking children between the ages of 2;6 and 9;11. We were also interested in examining differences among individual ICS items by child age. Toward this end, we addressed the following specific research questions: (a) How do ICS composite scores change as a function of age? and (b) How do the individual ICS item scores change as a function of age?

Based on the findings for Italian-speaking children aged 3;0–5;11 showing that ICS scores increased by .05 Likert points per month of age (Piazzalunga, Salerni, Ambrogi, et al., 2020), we hypothesize that ICS composite scores will show small increases with age. Similarly, on the basis of the findings for German-speaking children aged 3;0–5;11 showing positive correlations between most individual ICS item ratings and age (Neumann et al., 2017), we hypothesize that individual ICS item scores will increase with age but that the magnitude of increases may differ among ICS items.

## Method

This study was approved by the University of Wisconsin–Madison Institutional Review Board (Minimal Risk Research IRB Committee: 2016–0574). Informed consent was obtained on behalf of all participants. This study employed ICS data from the participants reported on by Hustad et al. (2020, 2021) and Mahr et al. (2021). We include a brief description of our methods in this article, but we refer interested readers to Hustad et al. (2021) for detailed information.

### Participants

Typically developing children from the Upper Midwest region of the United States were invited to participate in the study. To be included in the study, children met the following inclusion criteria: (a) chronological age was between 2;6 and 9;11; (b) primary language in the home was American English; (c) hearing was within normal limits as determined parent report, pure-tone hearing screening, or bilateral distortion product otoacoustic emission screening; (d) speech was within normal limits as determined by standardized scores on an articulation test (Fudala, 2003); and (e) language was within normal limits as determined by standardized scores on a language screener (Wiig et al., 2013; Zimmerman et al., 2012). Children were excluded if they had any medical diagnoses related to development or were receiving intervention services due to developmental and/or educational concerns.

Of the 573 typically developing children who qualified, 545 children were included in this study as their parents completed the ICS questionnaire. Note that all children who qualified were English monolingual speakers. Children included in the study ranged in age from 2;6 to 9;11. The sample comprised 281 girls and 264 boys. Note that 517 of these children are the same participants in the study by Hustad et al. (2021). Twenty-eight participants, for whom we did not have transcription intelligibility results at the time of publication for Hustad et al. (2021), were added to this study. Children's demographic data are presented in Table 1. Their age and sex distribution is shown in Table 2. Younger children were oversampled because of the variability demonstrated in an earlier study (Hustad et al., 2020). Moreover, Hustad et al. (2021) observed that the multiword intelligibility of children after 7 years of age was less varied and more homogeneous.

**Table 1.** Demographic information of typically developing children (*N* = 545).

Characteristic	Female (*n* = 281)	Male (*n* = 264)
Race		
White	248 (6^H^)	239 (9^H^, 2*)
Black	2	5
Asian	5	3
American Indian	1	0
Native Hawaiian/Pacific Islander	1	0
More than 1 race	22 (2^H^, 1*)	14
Other: pre/nonracial	2	
Not reported		3 (2^H^, 1*)
2-Factor Hollingshead Social Index mean^a^	55.34 (*SD* = 7.96)	55.50 (*SD* = 8.00)
Maternal education^b^		
Graduate professional training or graduate degree	140	123
Standard college or university graduation	114	122
Partial college or specialized training	22	11
High school graduate	4	6

*Note.* Within each racial category, the number of children whose parents identified them as having Hispanic ethnicity or preferred not to specify their ethnicity are denoted by superscripts “H” and “*,” respectively. All other children were identified by their parents as non-Hispanic.

a The 2-Factor Hollingshead Social Index mean of five participants was not determined as their parents were unemployed at the time of data collection.

b Three children had a single male parent or two male parents. Their data were not included in the summary of maternal education.

**Table 2.** Age and sex distribution of typically developing children.

Age range in years;months	Number of female	Number of male	Total	Mean age (*SD*)
2;6–2;11	26	27	53	2.73 (0.14)
3;0–3;5	25	24	49	3.20 (0.15)
3;6–3;11	34	20	54	3.71 (0.14)
4;0–4;5	35	17	52	4.20 (0.14)
4;6–4;11	25	29	54	4.73 (0.14)
5;0–5;5	21	27	48	5.22 (0.14)
5;6–5;11	24	27	51	5.71 (0.14)
6;0–6;5	20	24	44	6.22 (0.15)
6;6–6;11	27	28	55	6.68 (0.13)
7;0–7;5	14	13	27	7.18 (0.16)
7;6–7;11	11	7	18	7.68 (0.14)
8;0–8;5	6	3	9	8.21 (0.18)
8;6–8;11	9	2	11	8.76 (0.16)
9;0–9;5	1	7	8	9.19 (0.14)
9;6–9;11	3	9	12	9.68 (0.14)

### Materials and Procedure

*ICS ratings.* Parents of each child participant completed the ICS (McLeod et al., 2012) during the laboratory visit. When answering the seven ICS questions examining the child's ability to be understood by seven communicative partners who varied in familiarity (i.e., themselves, immediate family, extended family, friends, acquaintances, teachers, and strangers), parents were instructed to think about their child's speech with each communicative partner over the past month. Ratings were made using a 5-point ordinal scale, with 1 as the lowest score and 5 as the highest score. Following ICS published instructions (McLeod, 2012), an ICS composite score (i.e., overall intelligibility rating) was computed by averaging across the seven items. Data for each child participant included an ICS composite score and seven individual ICS item ratings. For the majority of the participants, their mother completed the ICS (*n* = 484; 90.64%). For the remaining participants, their father (*n* = 49; 8.99%) or their guardian (*n* = 2; 0.37%) completed the ICS.

*Statistical procedure*. In this study, we examined ICS composite scores and ICS individual item scores completed by parents or guardians. To answer the first research question on how ICS composite scores change as a function of age, ICS composite scores were regressed on age using a proportional odds model with a three-degree-of-freedom natural spline for age so that ICS composite scores could change flexibly with age. Model-estimated mean and quantile ICS composite scores were calculated to characterize the normative distribution of the ICS. Our analysis focused on lower quantiles (i.e., 5th, 10th, 25th, and 50th percentiles) as children in the upper quantiles achieved the ceiling score of 5 at earlier ages. Moreover, lower quantiles have more clinical relevance (e.g., determining children who are low performers or who may benefit from a comprehensive speech assessment).

To explore how the seven individual ICS item scores change as a function of age, descriptive statistics were used to quantify the changes in parent rating of each individual ICS item among children 2;6–9;11. Parents used only two unique ICS ratings (i.e., 4 and 5) for Item 1, resulting in a two-level outcome variable. Thus, a logistic regression with a three-degree-of-freedom natural spline for age was implemented for Item 1, so that scores could change flexibly with age. Probability and odds ratios were estimated from the logistic regression model. Parents used more than two ratings when responding to Items 2–7. Accordingly, a proportional odds model with a three-degree-of-freedom natural spline for age was implemented for Items 2–7, so that item scores could change flexibly with age. That is, individual ICS item scores were regressed on age separately.

Data analysis was executed in R (Version 4.2.1; R Core Team, 2021) and Rstudio (Version 2022.07.0; RStudio Team, 2021). Proportional odds modeling and logistic regression were completed using rms R package (Version 6.3-0; Harrell, 2022).

## Results

**Figure 1.**
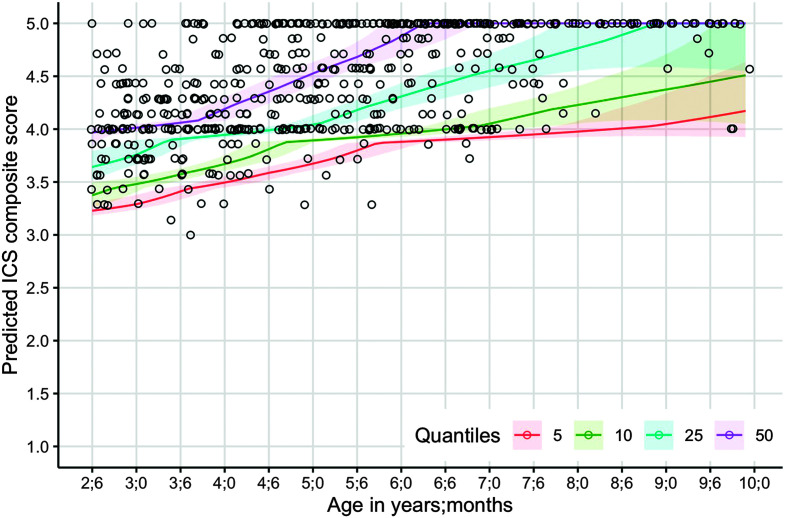
Model-estimated scores by percentile with observed data points from participants. ICS = Intelligibility in Context Scale.

*Research Question 1: How do ICS composite scores change as a function of age?* Figure 1 shows model-estimated ICS composite scores for 5th, 10th, 25th, and 50th percentiles of children. Using a proportional odds model with a three-degree-of-freedom natural spline to examine ICS composite scores as a function of age, the results of a likelihood ratio test showed a statistically significant relationship, χ^2^(3, *N* = 545) = 185.13, *p* < .001. That is, change in ICS composite scores was associated with change in age. However, data indicated that children younger than 6 years presented with wider score variability and a steeper growth curve as compared with older children. By 6 years of age, the majority of children received an ICS composite score of 5. When considering performance of children at different percentiles, results showed that the highest predicted ICS composite score of children in the 5th percentile was 4.14 around the age of 9;6. Meanwhile, the predicted ICS composite score of children in the 50th percentile reached the ceiling score of 5 just before the age of 6;6 years. See Tables 3 and 4 for summaries of the model-estimated mean ICS composite scores at half-year intervals and the model-estimated ICS composite scores at different percentiles (i.e., 5th, 10th, 25th, and 50th) and at half-year intervals, respectively.

**Table 3.** Model-estimated mean Intelligibility in Context Scale (ICS) composite scores by age of this study compared with mean ICS composite scores across specific age ranges of previous studies.

Age (years;months)	Soriano et al. (this study)	Age range (years;months)	Piazzalunga, Salerni, Ambrogi, et al. (2020)		Washington et al. (2017)	
			Mother ratings	Father ratings	English	Jamaican Creole
	*N* = 545		*n* = 327	*n* = 327	*n* = 145	*n* = 98
	*M* [95% CI]		*M* (*SD*)			
2;6	3.96 [3.84, 4.07]	2;6–2;11				
3;0	4.06 [3.99, 4.13]	3;0–3;5	4.26 (0.45)	4.14 (0.47)	4.34 (0.68)	4.42 (0.52)
3;6	4.17 [4.11, 4.22]	3;6–3;11	4.30 (0.47)	4.25 (0.58)		
4;0	4.27 [4.21, 4.34]	4;0–4;5	4.49 (0.50)	4.53 (0.46)		
4;6	4.38 [4.31, 4.45]	4;6–4;11	4.53 (0.45)	4.53 (0.49)		
5;0	4.48 [4.42, 4.54]	5;0–5;5	4.64 (0.40)	4.53 (0.42)	4.50 (0.58)	4.56 (0.56)
5;6	4.57 [4.51, 4.62]	5;6–5;11	4.72 (0.38)	4.67 (0.37)		
6;0	4.64 [4.58, 4.70]	6;0–6;5				
6;6	4.70 [4.64, 4.76]	6;6–6;11				
7;0	4.75 [4.69, 4.80]	7;0–7;5				
7;6	4.78 [4.73, 4.84]	7;6–7;11				
8;0	4.81 [4.76, 4.87]	8;0–8;5				
8;6	4.84 [4.77, 4.90]	8;6–8;11				
9;0	4.86 [4.78, 4.94]					
9;6	4.88 [4.79, 4.97]					

*Note.* Piazzalunga, Salerni, Ambrogi, et al. (2020) reported two sets of ICS composite scores: ratings from children's mother and ratings from children's father. Washington et al. (2017) reported two sets of ICS composite scores: parent rating on English intelligibility and parent rating on Jamaican Creole intelligibility.

**Table 4.** Model-estimated Intelligibility in Context Scale composite scores by percentile and age of this study compared with percentile scores reported by Piazzalunga, Salerni, Ambrogi, et al. (2020).

Age (years;months)	Soriano et al. (this study)	Age range (years;months)	Piazzalunga, Salerni, Ambrogi, et al. (2020)	
			Mother ratings	Father ratings
	*N* = 545		*n* = 327	*n* = 327
5th percentile				
2;6	3.23 [3.18, 3.31]	2;6–2;11		
3;0	3.29 [3.24, 3.38]	3;0–3;5	3.50	3.19
3;6	3.41 [3.33, 3.46]	3;6–3;11	3.43	3.29
4;0	3.49 [3.45, 3.55]	4;0–4;5	3.57	3.71
4;6	3.59 [3.52, 3.65]	4;6–4;11	3.86	3.66
5;0	3.67 [3.61, 3.75]	5;0–5;5	4.00	3.86
5;6	3.80 [3.70, 3.88]	5;6–5;11	3.86	3.99
6;0	3.88 [3.81, 3.90]	6;0–6;5		
6;6	3.90 [3.88, 3.93]	6;6–6;11		
7;0	3.92 [3.89, 3.96]	7;0–7;5		
7;6	3.95 [3.91, 4.00]	7;6–7;11		
8;0	3.98 [3.92, 4.08]	8;0–8;5		
8;6	4.01 [3.93, 4.20]	8;6–8;11		
9;0	4.05 [3.93, 4.34]			
9;6	4.11 [3.93, 4.50]			
10th percentile				
2;6	3.37 [3.26, 3.50]	2;6–2;11		
3;0	3.48 [3.41, 3.55]	3;0–3;5	3.71	3.43
3;6	3.57 [3.51, 3.63]	3;6–3;11	3.57	3.40
4;0	3.67 [3.61, 3.75]	4;0–4;5	3.77	3.86
4;6	3.81 [3.71, 3.89]	4;6–4;11	3.93	3.86
5;0	3.89 [3.87, 3.91]	5;0–5;5	4.00	4.00
5;6	3.92 [3.90, 3.95]	5;6–5;11	4.14	4.14
6;0	3.96 [3.93, 4.00]	6;0–6;5		
6;6	4.00 [3.95, 4.08]	6;6–6;11		
7;0	4.05 [3.98, 4.20]	7;0–7;5		
7;6	4.15 [4.02, 4.29]	7;6–7;11		
8;0	4.23 [4.05, 4.41]	8;0–8;5		
8;6	4.30 [4.08, 4.54]	8;6–8;11		
9;0	4.37 [4.08, 4.71]			
9;6	4.45 [4.07, 4.96]			
25th percentile				
2;6	3.64 [3.53, 3.79]	2;6–2;11		
3;0	3.76 [3.67, 3.90]	3;0–3;5	4.00	3.89
3;6	3.91 [3.83, 3.93]	3;6–3;11	4.00	3.86
4;0	3.95 [3.92, 3.97]	4;0–4;5	4.00	4.14
4;6	3.99 [3.96, 4.03]	4;6–4;11	4.14	4.14
5;0	4.04 [4.01, 4.13]	5;0–5;5	4.29	4.11
5;6	4.18 [4.07, 4.28]	5;6–5;11	4.43	4.43
6;0	4.31 [4.20, 4.44]	6;0–6;5		
6;6	4.43 [4.29, 4.58]	6;6–6;11		
7;0	4.55 [4.39, 4.71]	7;0–7;5		
7;6	4.65 [4.49, 4.87]	7;6–7;11		
8;0	4.76 [4.55, 5.00]	8;0–8;5		
8;6	4.89 [4.58, 5.00]	8;6–8;11		
9;0	5.00 [4.58, 5.00]			
9;6	5.00 [4.57, 5.00]			
50th percentile				
2;6	3.97 [3.84, 4.02]	2;6–2;11		
3;0	4.01 [3.98, 4.05]	3;0–3;5	4.14	4.14
3;6	4.06 [4.03, 4.12]	3;6–3;11	4.29	4.29
4;0	4.19 [4.08, 4.30]	4;0–4;5	4.57	4.57
4;6	4.35 [4.25, 4.48]	4;6–4;11	4.43	4.57
5;0	4.53 [4.41, 4.63]	5;0–5;5	4.79	4.57
5;6	4.68 [4.57, 4.82]	5;6–5;11	5.00	4.86
6;0	4.88 [4.70, 5.00]	6;0–6;5		
6;6	5.00 [4.85, 5.00]	6;6–6;11		
7;0	5.00 [5.00, 5.00]	7;0–7;5		
7;6	5.00 [5.00, 5.00]	7;6–7;11		
8;0	5.00 [5.00, 5.00]	8;0–8;5		
8;6	5.00 [5.00, 5.00]	8;6–8;11		
9;0	5.00 [5.00, 5.00]			
9;6	5.00 [5.00, 5.00]			

*Note.* Values in brackets indicate 95% confidence interval.

**Figure 2.**
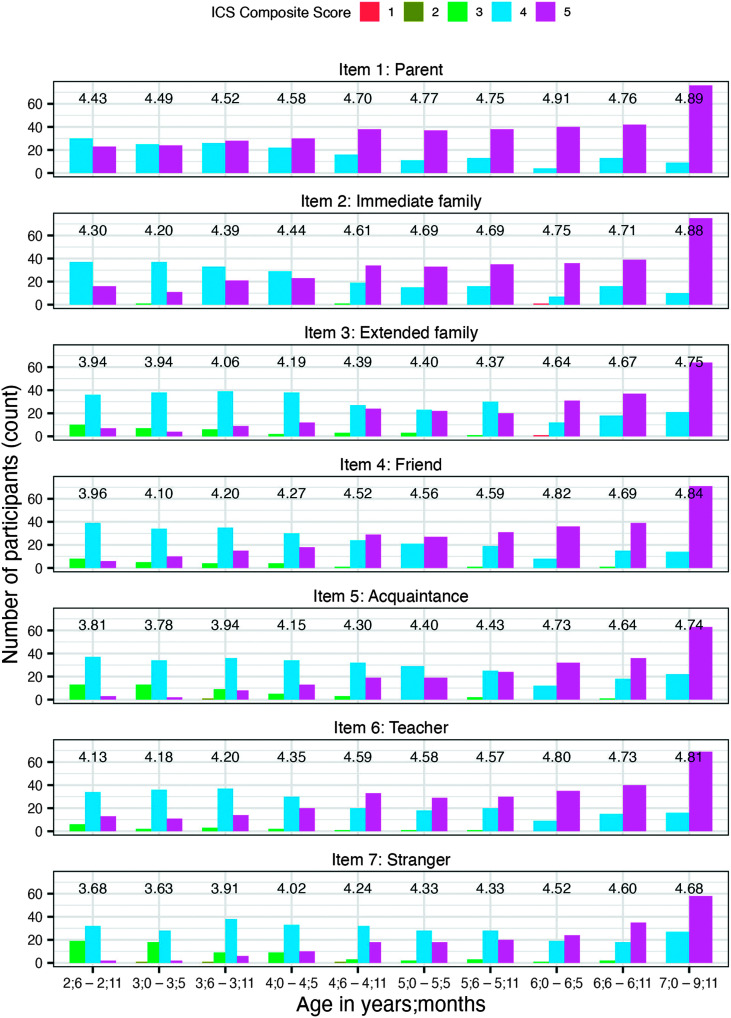
Frequency distribution of Intelligibility in Context Scale (ICS) scores by age and ICS individual question. Group means are noted for each age range and ICS item.

*Research Question 2: How do scores of individual ICS items change as a function of age?* Figure 2 shows the count of participants and mean scores grouped by individual ICS items and parent ratings. Note that a rating of 1 (i.e., *never intelligible*) was used only twice across all children and was observed for only two different questions (i.e., Item 2: “Do immediate members of your family understand your child?” for a child aged 6;4 years old and Item 3: “Do extended members of your family understand your child?” for the same child aged 6;4). Similarly, a rating of 2 (i.e., *rarely intelligible*) was used only 4 times across all children and was observed for only two different questions. Item 7 (i.e., “Do strangers understand your child?”) was rated as a 2 in three instances (i.e., children aged 3;0, 3;7, and 4;11); Item 5 (i.e., “Do other acquaintances understand your child?”) was rated as a 2 on one occasion (i.e., the same child aged 3;7 as for Item 7). Also, noteworthy was that Item 1 (i.e., “Do you understand your child?”) was rated as a 4 or a 5 by all parents (only two unique scale ratings).

**Figure 3.**
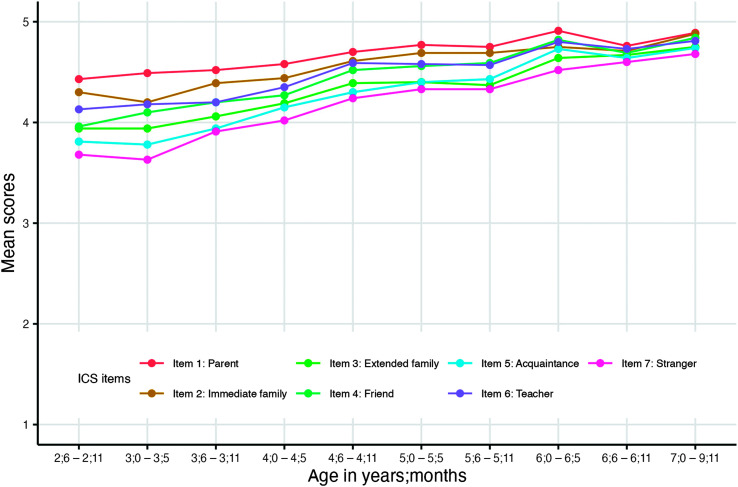
Mean scores of observed data grouped by individual Intelligibility in Context Scale (ICS) items.

Figure 3 shows the mean scores of observed data at specific age ranges grouped by individual ICS items. Between the ages of 2;6 and 9;11, parents consistently gave the highest rating to themselves and the lowest rating to stranger. Similarly, the ratings given to immediate family, teacher, and friend (familiar listeners) were higher than those given to extended family, acquaintance, and stranger (unfamiliar listeners). Moreover, differences between the ICS item mean scores decreased with age.

We examined the relationships between individual ICS items and age using a likelihood ratio test of the logistic regression (i.e., Item 1) and proportional odds model (i.e., Items 2–7). Table 5 summarizes the results of the likelihood ratio tests of each item. Similar to the ICS composite scores, all individual ICS scores changed as age changed.

**Table 5.** Likelihood ratio test.

ICS item	χ^2^(3, *N* = 545)	*p* value
Item 1	65.23	< .001
Item 2	105.05	< .001
Item 3	152.07	< .001
Item 4	151.29	< .001
Item 5	196.57	< .001
Item 6	116.56	< .001
Item 7	193.24	< .001

Item 1: Do you understand your child?

Item 2: Do immediate members of your family understand your child?

Item 3: Do extended members of your family understand your child?

Item 4: Do your child's friends understand your child?

Item 5: Do other acquaintances understand your child?

Item 6: Do your child's teacher understand your child?

Item 7: Do strangers understand your child?

*Note.* ICS = Intelligibility in Context Scale.

**Figure 4.**
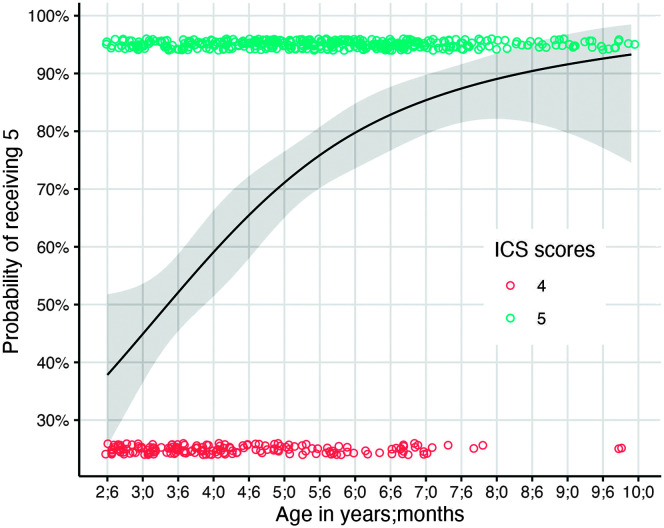
Probability of parents rating their child's intelligibility as 5 (i.e., *always understood*) with observed data points from participants. Data points at the top of the figure are participants who received a score of 5. Data points at the bottom of the figure are participants who received a score of 4. ICS = Intelligibility in Context Scale.

Figure 4 plots the probability of a child receiving a score of 5, that is, *always* understood by their parents (Item 1) across ages. Although the probability of receiving a score of 5 is higher than 50% by the age of 3;6 years, the odds ratio confidence interval included 1, indicating that children are similarly likely to receive scores of 4 or 5 at this age. However, by 4 years of age, typically developing children are 1.44 times more likely (with 59% probability) to be rated 5 or *always* understood by their parents.

For items specific to immediate family, friend, and teacher, starting at the age of 4;6 (see Figure 2), more parents rated their child 5 (i.e., always understood) than 4. However, for items specific to extended family, acquaintance, and stranger, it was not until the age of 6;0 that more parents rated their child 5 than 4.

## Discussion

In this study, we sought to quantify changes in ICS composite scores and individual ICS item scores as a function of age in typically developing children aged 2;6–9;11. The key findings were as follows: (a) ICS composite scores changed with age, but change was small and incremental, with scores compressed between 3 and 5 across the range of ages; (b) average children (50th percentile) had a model-estimated ICS composite score of 4 beginning at the age of 3;0, and a model-estimated ICS composite score of 5 by the age of 6;3; (c) model-estimated ICS composite scores of children in the lowest quantiles (i.e., 5th and 10th percentiles) ranged from 3.23 to 4.45 across the age range; and (d) on average, parents gave different intelligibility ratings based on communicative partners, but the mean differences between communicative partners decreased with age. These main findings are explored in detail below.

*How do ICS composite scores change as a function of age?* Our results aligned with the hypothesis that ICS composite scores of typically developing English-speaking children would increase by a small increment as age increased. The finding that ICS composite scores were compressed between 3 and 5 across the range of ages was generally consistent with studies that reported ICS composite scores on typically developing children who speak languages other than English.

Of the studies that reported mean ICS composite scores at specific age bands (see Appendix Table A2), only Piazzalunga, Salerni, Ambrogi, et al. (2020) and Washington et al. (2017) included typically developing children in their sample. The remaining studies included both typically developing children and children with speech-language disorders or whose parents had concerns about their speech production. Thus, this study was only comparable with that of Piazzalunga, Salerni, Ambrogi, et al. and Washington et al. in terms of mean ICS composite scores. Piazzalunga, Salerni, Ambrogi, et al. described mean ICS composite scores of monolingual Italian-speaking children aged 3;0–5;11 (*n* = 327), whereas Washington et al. described mean ICS composite scores of English–Jamaican Creole bilingual children aged 3;3–6;3 (*n* = 145). Although the age ranges between the studies were different, the model-estimated mean ICS composite scores from this study were comparable with the same-age mean ICS composite scores from the other two studies (see Table 3).

One interesting observation is that the results of this study and those of Washington et al. (2017) seem to suggest that speech intelligibility of monolingual English children and English–Jamaican Creole bilingual children for both of their languages falls within the same range in terms of age-based ICS scores. Given the variety of language experience and outcomes of bilingual children (Hoff, 2018), these findings may or may not apply to other bilingual/multilingual children. Research on the development of intelligibility in bilingual/multilingual children is an area in dire need of attention.

Of the three studies that reported on ICS composite scores for specific percentiles, only Piazzalunga, Salerni, Ambrogi, et al. (2020) included typically developing children in their sample. For both studies, the estimated mean ICS composite scores of children aged 3;0–5;11 were between 4 and 5 (of 5). However, model-estimated mean ICS composite scores from this study were consistently lower than the mean ICS composite scores reported by Piazzalunga, Salerni, Ambrogi, et al. across the different age ranges (see Table 3). This pattern was similar for ICS composite percentile scores. Across different percentiles (see Table 4), model-estimated ICS composite scores from this study were generally consistent but generally lower than the ICS composite scores reported by Piazzalunga, Salerni, Ambrogi, et al. The magnitude of differences between the two studies was more prominent for older age ranges and higher quantiles (i.e., 25th and 50th percentiles). A common trend was that Italian-speaking children started to receive higher ICS composite scores relatively earlier than English-speaking children.

Results of this study and that of Piazzalunga, Salerni, Ambrogi, et al. (2020) suggest that intelligibility ratings of English- and Italian-speaking children are comparable but not identical. These differences may be associated with variations in language and/or cultural perspectives on speech intelligibility. It may be the case that the parents of English-speaking children have more difficulty understanding their children as compared with parents of Italian-speaking children. Another possibility is that parents of Italian-speaking children overestimate their child's intelligibility. More cross-linguistic studies on speech intelligibility are needed to explore these differences.

In general, this study on English and previous studies from other languages indicate that ICS overall intelligibility ratings (i.e., average ratings across different communicative partners; composite scores) of typically developing children between the ages of 2;6 and 10;5 can range from *sometimes understood* (rating of 3) to *always understood* (rating of 5). Children in the lowest quantiles (i.e., 5th and 10th percentiles) were reported to have ICS composite scores between *sometimes understood* (rating of 3) and *usually understood* (rating of 4). Across this study and previous studies, none of the children received an overall intelligibility rating of *rarely understood* (rating of 2) or *never understood* (rating of 1). Clinically, this may indicate that children who receive ICS composite scores between 1 and 2 (regardless of age) warrant a comprehensive speech evaluation. Children aged 6 years and older are expected to receive an ICS composite score of at least 4 or higher (see Figure 1). In other words, older children who receive an ICS composite score of 3 may benefit from a comprehensive speech evaluation, whereas children younger than 6 years old who receive an ICS composite score of 3 or 4 can benefit from close monitoring.

We highlight here that findings in this study indicating that ratings below 3 were not utilized, even for younger children, differ from results based on transcription intelligibility. Specifically in research employing orthographic transcription methods, some younger children had very low intelligibility scores (Hustad et al., 2021). The difference between intelligibility development findings from the two measurement methods suggests that contextually based ratings from the ICS may be a more generous reflection of intelligibility. The relationship between ICS scores and transcription intelligibility has not been investigated in typically developing children; this is an important avenue for future research.

*How do scores of individual ICS items change as a function of age?* Our hypothesis that individual ICS item scores would increase with age, but the pattern of increase would be different across ICS items, was consistent with the results. Across ages, parents gave the highest rating to themselves and the lowest to strangers. Similar findings have been noted across studies on the ICS (McLeod, 2020). We also found that differences between communicative partners decreased with age (see Figure 3). The observation that the differences between mean intelligibility ratings of each communicative partner decreased over time is consistent with findings of Flipsen (1995), who examined four children with speech delay and reported that the maternal advantage on understanding a child's speech was less for older participants. The decrease in the difference between intelligibility ratings of communicative partners with varying familiarity is a natural consequence of the developmental nature of speech intelligibility. That is, as children develop more accurate and clear speech, the advantage of being a familiar listener (e.g., parent) on understanding a child's speech decreases. Thus, the rating differences between familiar and unfamiliar listeners would be expected to decrease. Specific to this study, the majority of parents of typically developing children rated their child's speech as *always understood* (rating of 5) by themselves starting at the age of 4;0 (see Figure 2). Starting at the age of 4;6, the majority of parents rated their child's speech as *always understood* (rating of 5) by immediate family, friend, and teacher. Starting at the age of 6;0, the majority of parents rated their child's speech as *always understood* (rating of 5) by extended family, acquaintance, and stranger. This can be interpreted that the advantage of being a familiar listener on understanding a child's speech is diminished by the age of 6;0. Previous studies have described the disadvantages of rating scales, such as overestimation or underestimation of actual performance (Kent et al., 1994; Miller, 2013; Schiavetti, 1992). To validate the hypothesis that the advantage of being a familiar listener on understanding a child's speech diminishes by the age of 6;0, studies that compare the intelligibility differences between parents and unfamiliar listeners using objective measures such as orthographic transcription are needed.

### Clinical Implications

The clinical advantages of using the ICS are ease; speed of administration; and the ability to obtain an overall intelligibility rating across different communication partners (i.e., composite score) while, at the same time, providing ratings for each specific type of communication partner. Results of this study on typically developing children indicate that children who receive ICS composite scores of 2 or lower, regardless of age, should be referred for a comprehensive speech evaluation. For children who receive ICS composite scores of 3 to 4, the decision of whether to refer for speech evaluation depends on the child's age. That is, children younger than 6 years (who have ICS composite scores of 3 to 4) fall into the lowest quantiles of typical expectations. They may benefit from monitoring. Children older than 6 years are expected to have an ICS composite score of at least 4. Thus, older children who receive an ICS composite score below 4 should be referred for a comprehensive speech evaluation.

Similar to other studies on the ICS, this study found that parents gave different ratings for each communication partner, allowing clinicians' insight into a child's speech performance in different contexts. When the ICS is combined with other methods for measuring speech intelligibility, clinicians can put together a better depiction of a child's speech abilities.

### Limitations and Future Directions

This study expanded the literature on the developmental context of speech intelligibility, specifically adding normative data for English-speaking typically developing children for the ICS. However, this study utilized a cross-sectional data set that limits the interpretability of growth/development. Longitudinal studies that include children who are typically developing and those with speech disorders are necessary to advance our understanding of within-child growth patterns and to enable us to understand how parent ratings of speech intelligibility differ for children with different speech characteristics.

The current data set included children from the Upper Midwest region of the United States who used American English as their primary language. They were relatively homogeneous with regard to race and ethnicity and tended to be from highly educated families. Normative studies are needed that examine children from other regions of the United States, including children from a wide array of different dialect, race, and ethnic groups; children who are bilingual/multilingual; and children from families that vary in socioeconomic backgrounds. In addition, normative studies from other regions of the world are also needed to advance our understanding of how language and culture impact parents' perception of speech intelligibility.

This study examined age-related change in ICS scores; however, we did not examine how ICS scores may predict or explain intelligibility as measured using objective measures such as orthographic transcription. Studies are needed that examine the extent to which ICS scores may or may not reflect other measures of speech intelligibility.

Differences of ICS scores between girls and boys were not considered during the analysis. Normative studies that explore the effects of sex on ICS scores are needed as they can inform clinicians of expected differences in performance between boys and girls.

## Data Availability Statement

The data used for the analyses presented in this study may be made available upon request. The data are not publicly available due to human subjects' privacy restrictions.

## Appendix

### Summary of Intelligibility in Context Scale Scores Reported by Previous Studies

**Table A1.** Summary of mean Intelligibility in Context Scale (ICS) composite scores reported by available literature.

Article	Language assessed	Version of ICS used	Sample			Mean composite score (*SD*)		
			Age range (years;months)	Speech-language status	Language use	Typically developing children	Children with parental concerns and/or disorder	All
Hopf et al. (2017)	Main language	English	5;3–10;5	Typically developing	Multilingual	**4.6 (0.6)** ***n* = 65**	—	—
	Fiji English	English	5;3–10;5	Typically developing or with parental concerns	Multilingual	**—**	—	4.4 (0.5) *N* = 52
Kim et al. (2016)	English–Korean bilingual	Korean	3;0–5;11	Typically developing	Bilingual	**4.50 (0.39)** ***n* = 20**	4.39 (0.45) *N* = 13	4.4 (0.42) *N* = 33
Kok & To, (2019)	Cantonese	Traditional Chinese	2;4–6;9	Typically developing or with speech sound disorders	Monolingual	**4.03 (0.70)** ***n* = 642**	3.63 (0.66) *N* = 147	3.96 (0.71) *N* = 789
Lagerberg et al. (2021)	Swedish	Swedish	3;2–9;2	Typically developing or with parental concerns	Monolingual	**4.81 (0.39)** ***n* = 266**	4.28 (0.54) *N* = 53	4.73 (0.4) *N* = 319
Le et al. (2021)	Vietnamese	Vietnamese	3;11–5;11	Typically developing	Monolingual	**4.55 (0.54)** ***n* = 132**	—	—
Lee (2019)	Korean	Korean	2;6–6;5	Typically developing or with speech sound disorders	Monolingual	**4.64 (0.41)** ***n* = 145**	3.67 (0.62) *N* = 33	4.46 (0.59) *N* = 178
Leon et al. (2021)	English–JC simultaneous bilingual	English	3;3–6;3	Typically developing or with speech sound disorders	Bilingual	**4.57 (0.58)** ***n* = 237**	3.66 (0.71) *N* = 25	4.48 (0.65) *N* = 262
	English–JC simultaneous bilingual	Jamaican Creole	3;3–6;3	Typically developing or with speech sound disorders	Bilingual	**4.59 (0.54)** ***n* = 190**	3.71 (0.70) *N* = 25	4.48 (0.63) *N* = 215
Lousada et al. (2019)	European Portuguese	European Portuguese	3;10–6;2	Typically developing or with parental concerns	Monolingual	**4.78 (0.36)** ***n* = 51**	3.91 (0.59) *N* = 25	4.49 (0.60) *N* = 76
McLeod et al. (2015)	Australian English	English	4;0–5;5	Typically developing, with parental concerns, or with speech sound disorders	Monolingual and multilingual	**4.6 (0.5)** ***n* = 108**	3.9 (*SD* not reported) *N* = 623	4.4 (0.7) *N* = 803
McLeod et al. (2012)	Australian English	English	3;11–5;8	Typically developing or with parental concerns	Monolingual and multilingual	**4.69 (0.51)** ***n* = 11**	3.85 (0.30) *N* = 109	—
Neumann et al. (2017)	German	German	3;0–5;11	Typically developing or with speech sound disorders	Monolingual and bilingual	**4.49 (0.47)** ***n* = 151**	3.97 (0.62) *N* = 30	4.40 (0.53) *N* = 181
Ng et al. (2014)	Cantonese	Traditional Chinese	3;0–6;0	Typically developing or with speech sound disorders	Monolingual	**4.56 (0.48)** ***n* = 39**	4.14 (0.65) *N* = 33	—
Phạm et al. (2017)	Vietnamese	Vietnamese	2;0–5;11	Typically developing or with parental concerns	Monolingual	**4.63 (0.67)** ***n* = 148**	4.29 (0.67): little concern *N* = 43 3.96 (0.70): yes, have concern *N* = 33	4.43 (0.62) *N* = 181
Piazzalunga, Salerni, Limarzi, et al. (2020)	Italian	Italian - completed by mothers	3;0–5;11	Typically developing	Monolingual and multilingual	**4.52 (0.46)** ***n* = 352**	—	—
	Italian	Italian -completed by fathers	3;0–5;11	Typically developing	Monolingual and multilingual	**4.47 (0.49)** ***n* = 350**	—	—
Piazzalunga, Salerni, Limarzi, et al. (2020)	Italian	Italian - completed by mothers	3;0–5;11	Typically developing	Monolingual and multilingual	**4.51 (0.47)** ***n* = 361**	—	—
	Italian	Italian - completed by fathers	3;0–5;11	Typically developing	Monolingual and multilingual	**4.46 (0.50)** ***n* = 358**	—	—
Washington et al. (2017)	English	English	3;3–6;3	Typically developing	Bilingual	**4.43 (0.63)** ***n* = 145**	—	—
	Jamaican Creole	Jamaican Creole	3;3–6;3	Typically developing	Bilingual	**4.50 (0.57)** ***n* = 98**	—	—

*Note.* JC = Jamaican Creole.

**Table A2.** Summary of mean Intelligibility in Context Scale composite scores reported by available literature at specific age bands.

Age range in years;months	Kok & To (2019)	Lagerberg et al. (2021)	Lousada et al. (2019)	McLeod et al. (2015)	Phạm et al. (2017)	Piazzalunga, Salerni, Ambrogi, et al. (2020)		Washington et al. (2017)	
						Mother ratings	Father ratings	English	Jamaican Creole
	*N* = 789	*N* = 319	*N* = 76	*N* = 803	*N* = 181	*n* = 327	*n* = 327	*n* = 145	*n* = 98
2;0–2;5					3.96 (0.77)				
2;6–2;11	3.76 (0.64)				4.19 (0.79)				
3;0–3;5	3.68 (0.69)				4.19 (0.56)	4.26 (0.45)	4.14 (0.47)	4.34 (0.68)	4.42 (0.52)
3;6–3;11	3.66 (0.70)		4.11 (0.70)		4.45 (0.47)	4.30 (0.47)	4.25 (0.58)		
4;0–4;5	3.92 (0.82)	4.63 (0.40)		4.30 (0.70)	4.43 (0.71)	4.49 (0.50)	4.53 (0.46)		
4;6–4;11	4.12 (0.59)		4.69 (0.51)	4.40 (0.60)	4.58 (0.51)	4.53 (0.45)	4.53 (0.49)		
5;0–5;5	4.19 (0.65)	4.78 (0.30)		4.30 (0.70)	4.66 (0.48)	4.64 (0.40)	4.53 (0.42)	4.50 (0.58)	4.56 (0.56)
5;6–5;11	4.28 (0.57)		4.60 (0.47)			4.72 (0.38)	4.67 (0.37)		
6;0–6;5	4.25 (0.58)	4.80 (0.30)							
6;6–6;11									
7;0–7;5		4.71 (0.50)							
7;6–7;11									
8;0–8;5		4.74 (0.48)							
8;6–8;11									

*Note.* Values enclosed in parentheses are reported standard deviations. The studies by Kok and To (2019) on Cantonese-speaking children, Lagerberg et al. (2021) on Swedish-speaking children, Lousada et al. (2019) on European Portuguese–speaking children, McLeod et al. (2015) on Australian English–speaking children, and Phạm et al. (2017) on Vietnamese-speaking children included typically developing children and children with speech-language disorders or whose parents have concerns on their speech production. The studies by Piazzalunga, Salerni, Ambrogi, et al. (2020) on Italian-speaking children and Washington et al. (2017) on Jamaican Creole–English bilingual speaking children included only typically developing children. Piazzalunga, Salerni, Ambrogi, et al. (2020) reported two sets of Intelligibility in Context Scale (ICS) composite scores: ratings from children's mother and ratings from children's father. Washington et al. (2017) reported two sets of Intelligibility in Context Scale (ICS) composite scores: parent rating on English intelligibility and parent rating on Jamaican Creole intelligibility.

**Table A3.** Summary of Intelligibility in Context Scale composite scores reported by available literature at percentiles.

Age range (years;months)	Lagerberg et al. (2021)	Lousada et al. (2019)	Piazzalunga, Salerni, Ambrogi, et al. (2020)	
			Mother ratings	Father ratings
	5th percentile			
3;0–3;5			3.50	3.19
3;6–3;11			3.43	3.29
4;0–4;5	3.69		3.57	3.71
4;6–4;11			3.86	3.66
5;0–5;5	3.91		4.00	3.86
5;6–5;11			3.86	3.99
6;0–6;5	3.85			
6;6–6;11				
7;0–7;5	3.57			
7;6–7;11				
8;0–8;5	3.48			
8;6–8;11				
	10th percentile			
3;0–3;5			3.71	3.43
3;6–3;11			3.57	3.40
4;0–4;5	4.00		3.77	3.86
4;6–4;11			3.93	3.86
5;0–5;5	4.00		4.00	4.00
5;6–5;11			4.14	4.14
6;0–6;5	4.00			
6;6–6;11				
7;0–7;5	3.86			
7;6–7;11				
8;0–8;5	3.82			
8;6–8;11				
	15th percentile			
3;0–3;5			3.86	3.62
3;6–3;11			3.71	3.57
4;0–4;5			4.00	3.90
4;6–4;11			4.00	4.00
5;0–5;5			4.00	4.00
5;6–5;11			4.06	4.24
	25th percentile			
3;0–3;5			4.00	3.89
3;6–3;11		3.40	4.00	3.86
4;0–4;5	4.17		4.00	4.14
4;6–4;11		4.57	4.14	4.14
5;0–5;5	4.85		4.29	4.11
5;6–5;11		4.25	4.43	4.43
6;0–6;5	4.85			
6;6–6;11				
7;0–7;5	4.42			
7;6–7;11				
8;0–8;5	4.71			
8;6–8;11				
	50th percentile			
3;0–3;5			4.14	4.14
3;6–3;11		4.14	4.29	4.29
4;0–4;5	5.00		4.57	4.57
4;6–4;11		5.00	4.43	4.57
5;0–5;5	5.00		4.79	4.57
5;6–5;11		4.93	5.00	4.86
6;0–6;5	5.00			
6;6–6;11				
7;0–7;5	5.00			
7;6–7;11				
8;0–8;5	5.00			
8;6–8;11				
	75th percentile			
3;0–3;5			4.57	4.43
3;6–3;11		5.00	4.57	4.71
4;0–4;5			5.00	5.00
4;6–4;11		5.00	5.00	5.00
5;0–5;5			5.00	5.00
5;6–5;11		5.00	5.00	5.00
6;0–6;5				
	90th percentile			
3;0–3;5			5.00	4.86
3;6–3;11			5.00	5.00
4;0–4;5			5.00	5.00
4;6–4;11			5.00	5.00
5;0–5;5			5.00	5.00
5;6–5;11			5.00	5.00
	95th percentile			
3;0–3;5			5.00	4.94
3;6–3;11			5.00	5.00
4;0–4;5			5.00	5.00
4;6–4;11			5.00	5.00
5;0–5;5			5.00	5.00
5;6–5;11			5.00	5.00

*Note.* The studies by Lagerberg et al. (2021) on Swedish-speaking children and Lousada et al. (2019) on European Portuguese–speaking children included typically developing children and children with speech-language disorders or whose parents have concerns on their speech production. The study by Piazzalunga, Salerni, Ambrogi, et al. (2020) on Italian-speaking children included only typically developing children and reported two sets of Intelligibility in Context Scale composite scores: ratings from children's mother and ratings from children's father.
